# Consumer Risk Characterisation of Potentially Toxic Elements in European Seabass (*Dicentrarchus labrax*) Marketed in Hungary

**DOI:** 10.3390/toxics14090767

**Published:** 2026-08-27

**Authors:** József Lehel, Keisuke Machida, András Bartha, Péter Palotás, Attila László Nagy, Orsolya Strang, Zsuzsa Farkas

**Affiliations:** 1 Department of Food Hygiene, Institute of Food Chain Science, University of Veterinary Medicine Budapest, István u. 2, H-1400 Budapest, Hungary; lehel.jozsef@univet.hu (J.L.); k516mac10@googlemail.com (K.M.); 2 Department of Animal Hygiene, Herd Health and Mobile Clinic, University of Veterinary Medicine Budapest, István u. 2, H-1400 Budapest, Hungary; bartha1952@gmail.com; 3 Department of Refrigeration and Livestock Products Technology, Hungarian University of Agriculture and Life Science, H-1118 Budapest, Hungary; peter.palotas72@gmail.com; 4 Institute of Food Chain Science, University of Veterinary Medicine Budapest, István u. 2, H-1078 Budapest, Hungary; nagy.attila.laszlo@univet.hu; 5 Department of Digital Food Science, Institute of Food Chain Science, University of Veterinary Medicine Budapest, István u. 2, H-1078 Budapest, Hungary; farkas.zsuzsa@univet.hu

**Keywords:** potentially toxic elements, arsenic, cadmium, lead, mercury, European seabass, dietary exposure, consumer risk assessment

## Abstract

European seabass (*Dicentrarchus labrax*) is a widely consumed fish species and may contribute to dietary exposure to potentially toxic elements. This study analysed 40 seabass samples, originating from the Adriatic Sea (Croatia) and purchased at retail in Hungary, for total arsenic (As), cadmium (Cd), lead (Pb), and mercury (Hg) concentrations using ICP-OES. Their concentrations in muscle tissue (mg/kg ww) were 1.44 ± 0.90 for total As (detected in 92.5% of samples), 0.06 ± 0.02 for Cd (detected in 35.0% of samples), and 0.25 ± 0.14 for Pb (detected in 27.5% of samples). Inorganic arsenic (iAs) was not measured directly but was conservatively estimated to be 5% of the total arsenic, resulting in an estimated concentration of 0.07 ± 0.05 mg/kg wet weight. All Hg results were below the analytical limit of detection (0.50 mg/kg). When compared with the EU maximum levels (ML), six samples (15%) exceeded the ML for Pb (0.30 mg/kg), thirteen samples (32.5%) exceeded the ML for Cd (0.05 mg/kg), and eight samples (20%) potentially exceeded the ML for iAs (0.1 mg/kg). Consumer risk was evaluated using a screening-level non-cancer risk characterisation framework based on the target hazard quotient (THQ) and hazard index (HI). Results below the LODs were handled using lower-, middle- and upper-bound substitution scenarios. Risk characterisation focused on EU-relevant consumption scenarios, including average adult consumptions, an adult high-consumer (P95) scenario, and child-specific scenarios using age-appropriate body weights. For average adult consumptions, the HI was below one under the lower-bound scenario but exceeded one under the middle- and upper-bound scenarios. However, under high-consumption conditions, lead was identified as the dominant contributor to elevated THQ and HI values, particularly in upper-bound scenarios and for sensitive population groups. These findings indicate that European seabass does not provide definitive evidence of a general population-level health risk; however, screening-level non-cancer concerns were identified under the mean adult middle- and upper-bound scenarios, as well as under high-consumption scenarios, underscoring the importance of continued monitoring and risk-based communication.

## 1. Introduction

Fish and seafood are important components of the human diet worldwide, valued not only for their high-quality protein content but also for their abundance of essential nutrients. Over the past 50 years, global fish and seafood consumption has more than doubled due to globalisation, population growth, and increasing awareness of the nutritional benefits of aquatic foods [1]. The world production of seabass almost doubled between 2014 and 2021 before stabilising around 290,000 tonnes in 2023. With its production exceeding 86,500 tonnes in 2023, the European Union (EU) accounted for 30% of the global production of seabass. According to the latest EU market report, Croatian aquaculture seabass production reached 8515 tonnes in 2023 [2]. In coastal regions and areas with easy access to marine or freshwater resources, fish and seafood play an important dietary and cultural role, often forming the basis of traditional cuisines, including raw or minimally processed dishes. However, alongside their nutritional benefits, fish and seafood may also pose risks due to contamination by chemical pollutants. Populations with high fish and seafood intakes may therefore be disproportionately exposed to these contaminants [3,4,5].

Potentially toxic elements (PTEs), including arsenic (As), cadmium (Cd), lead (Pb), and mercury (Hg), represent an important group of contaminants affecting aquatic environments. Although they occur naturally, industrial activities such as mining, smelting, manufacturing, and improper waste disposal can substantially increase their environmental concentrations. Once released, these elements can contaminate surface waters, sediments, and biota, entering the aquatic food chains and undergoing bioconcentration and, in some cases, biomagnification. Some aquatic organisms, such as molluscs, can detoxify metals through metallothionein production, but this process also leads to metal accumulation in edible tissues [6,7,8,9].

Fish can absorb PTEs not only through their diet but also from water across the gills and through their skin. Therefore, it is crucial to regulate the levels of these contaminants in fish for human consumption [10,11,12,13]. While trace amounts of certain elements (e.g., copper (Cu), zinc (Zn), manganese (Mn), nickel (Ni)) are essential for human physiology, their excessive intake can disrupt biological functions. In contrast, potentially toxic elements, such as mercury (Hg), cadmium (Cd), lead (Pb), and arsenic (As), have no beneficial role and may cause adverse health effects following chronic dietary exposure, particularly when bioaccumulated in seafood consumed by humans [12,14,15,16]. Mercury, primarily emitted from natural geological sources and anthropogenic activities, is deposited into aquatic systems where it can be transformed into methylmercury, which is a potent neurotoxic metal. Atmospheric deposition is the dominant source of mercury entering the oceans, and industrialisation has significantly increased global mercury levels. Methylmercury bioaccumulates efficiently and biomagnifies through marine food webs, reaching high concentrations in predatory fish. Its transfer from phytoplankton to zooplankton is far more efficient than that of inorganic mercury, and planktivorous fish accumulate methylmercury at levels up to 16 times higher than inorganic Hg [17,18,19]. Chronic exposure in humans is associated with sensory disturbances, visual field constriction, impaired muscle movements, and muscle weakness due to pathological (e.g., oedema, demyelination) and histopathological changes in the cerebrum, cerebellum, and peripheral nerves [20].

Cadmium enters aquatic ecosystems through natural processes and industrial emissions (e.g., metal industry, mining, smelting) [21]. After atmospheric deposition, Cd accumulates in soils and water bodies where it can be taken up by plants and aquatic organisms. Several seafood species, including crustaceans, molluscs, shellfish, and fish, are considered additional sources of Cd in the human diet, and some of them are also known as hyper-accumulators [22]. In fish such as carp (*Cyprinus carpio*), Cd accumulates primarily in the kidney and liver, with lower levels in muscle tissue [23]. Cd exposure is linked to carcinogenicity, oxidative stress [24,25], cytotoxicity, and endocrine-disrupting effects [26].

Lead remains a persistent environmental contaminant, originating from toys, paints, and other industrial products despite regulatory restrictions. It is widely distributed in the atmosphere, water, dust, and soil, and thus, in food, particularly in urban and industrialised regions [27,28]. Pb is non-biodegradable and can cause severe neurological, hematopoietic, hepatic, and renal toxicity, with no known safe exposure threshold for humans [28,29,30].

Arsenic occurs in the environment naturally and due to anthropogenic activities in both inorganic and organic forms. Inorganic arsenic (iAs), such as arsenites and arsenates, is more toxic than organic arsenic species and has a carcinogenic effect. While seafood predominantly contains organic arsenic species such as arsenobetaine and arsenocholine, which are considered relatively non-toxic, seafood may also contribute to iAs exposure depending on the species, tissue type, and arsenic speciation profile [31,32,33]. Arsenic exposure occurs primarily through contaminated drinking water, certain foods, or industrial inhalation. Acute toxicity causes gastrointestinal symptoms, while chronic exposure is associated with carcinogenic, cardiovascular, dermatological, and neurotoxic effects [34,35,36].

To protect consumers, the European Union regulates the maximum levels (MLs) of Pb, Cd, and Hg under Regulation (EU) 2023/915 and of iAS under Regulation (EU) 2025/1891 in fish; ML values vary by species and element, reflecting differences in their bioaccumulation potential [37,38].

Beyond compliance, dietary risk characterisation commonly combines occurrence data with consumption assumptions to estimate dietary exposure (estimated daily intake, EDI) and non-cancer risk characterisation metrics, such as the target hazard quotient (THQ) and hazard index (HI) [21,39,40,41]. However, risk estimates can be sensitive to (i) the treatment of measurements below the analytical limits of detection, and (ii) the choice of consumption and body-weight assumptions, particularly for children and high consumers. Unlike previous studies primarily reporting contaminant occurrence, the present study combines analytical determinations with a transparent screening-level consumer risk characterisation using lower-, middle-, and upper-bound scenarios; age-specific exposure assessments; and high-consumer scenarios to better capture uncertainty in dietary exposure estimates.

The objective of this study was to quantify the selected potentially toxic elements in European seabass (*Dicentrarchus labrax*) originating from the Adriatic Sea and retailed in Hungary, and to provide a transparent dietary exposure and non-cancer risk characterisation for EU consumers. Specifically, the study applied measurements below the analytical limits of detection and evaluated average- and high-consumption scenarios for both adults and children to support a transparent screening-level interpretation of consumer risk.

## 2. Materials and Methods

### 2.1. Samples and Study Design

The samples of European seabass (*Dicentrarchus labrax*) were collected during a single sampling campaign in March 2023 from the Budaörs Fish Market (Budaörs, Hungary), though the fish originated from the Central Mediterranean region (FAO Fishing Area, Subarea 37.2), including the Adriatic Sea (FAO Fishing Area, Division 37.2.1) [42]. A total of 40 fish were examined to determine their potentially toxic element concentrations, including arsenic, cadmium, lead, and mercury. As the study was designed as a retail-based consumer exposure assessment, the biological characteristics (e.g., fish weight, length, and age) and production-related information (e.g., farming status or capture method) were not included in the sampling protocol. The available traceability information was limited to the product label.

### 2.2. Methods

#### 2.2.1. Analytical Process

##### Reagents and Analytical Standards

For the preparation of samples, hydrogen peroxide (30% *w*/*w*, Normapur, VWR International Ltd., Leicestershire, UK) and nitric acid (69% *w*/*w*, Aristar, VWR International Ltd., Leicestershire, UK) were used, both of trace analysis quality. To clean all laboratory glassware and plastic tools, a 0.15 M hydrochloric acid solution (37% *w*/*w*, Aristar, VWR International Ltd., Leicestershire, UK) was employed, followed by rinsing with deionized water generated by a Purite Select Fusion 160 BP water purification system (Suez Water Ltd., Thame, UK). Calibration was carried out using ICP multi-element standards (Perkin Elmer Inc., Shelton, CT, USA) and mono-element standards (VWR International Ltd., Leicestershire, UK) for quantitative ICP measurement. Quality control (QC) standards were prepared from standard bovine liver (NIST SRM 1577c, NIST, Gaithersburg, MA, USA), and measurements utilised argon gas with a purity of 4.6 (Messer Hungarogáz Ltd., Budapest, Hungary).

##### Sample Preparation

A metal-free instrument was employed to section the longitudinal back muscle samples from European seabass. Briefly, dorsal muscle samples were excised using metal-free instruments. Following cutting and homogenization using a Potter S device (B. Braun Biotech International GmbH, Melsungen, Germany), the prepared samples were placed in appropriately labelled plastic bags and stored at –70 °C in a So-Low Ultra-Low Freezer (Model C85-9, Environmental Equipment Co. Inc., Cincinnati, OH, USA) until analysis.

For each sample, precisely 0.5 g was weighed into a CEM MARS XPreSS Teflon vessel (CEM, Matthews, NC, USA). Hydrogen peroxide and nitric acid were added at 5 mL each, and microwave digestion was performed in a CEM MARS6 microwave digestion system (CEM Corporation, Matthews, NC, USA). The digestion parameters were as follows: ramp time, 35 min; temperature, 200 °C; hold time, 50 min; and power, 1700 W. The resulting solution was adjusted to 25 mL with deionized water, and ICP-OES was employed for analysis following a two-fold dilution with deionized water. A 1 mg/L Y solution (VWR International Ltd., Leicestershire, UK) served as an internal standard, and a 0.25 mg/L Au solution (VWR International Ltd., Leicestershire, UK) was used for mercury stabilisation. The preparation of blank and quality control (QC) samples followed the same procedure.

##### Instrumentation

The analysis of PTEs was conducted using a Perkin Elmer Optima 8300 DV (Perkin Elmer, Shelton, CT, USA) Inductively Coupled Plasma Optical Emission Spectrometer (ICP-OES) instrument, with the following measurement parameters: RF generator—40 MHz solid-state, flat plate plasma technology, and free running; RF power—1300 W; nebulizer type (Burgener PEEK Mira Mist (Thermo Fisher Scientific, Budapest, Hungary)); plasma gas flow rate—12 dm^3^/min; nebulizer gas flow rate—0.7 dm^3^/min; auxiliary gas flow rate—0.2 dm^3^/min; and observation height—15 mm. The wavelengths detected for each element are presented in Table 1.

#### 2.2.2. Validation of the Analytical Process

To evaluate the effectiveness of sample preparation and the reliability of the analytical method, several validation parameters were determined in accordance with relevant guidelines, as shown in Table 1 [43].

Limits of detection (LODs) and limits of quantitation (LOQs) were determined as three and ten times the standard deviation of the signals from blank samples, respectively. Precision was assessed as the relative standard deviation of signals obtained from ten replicates of the same sample. Trueness was evaluated using certified reference materials (NIST SRM 1577c bovine liver, Merck Life Science Kft., Budapest, Hungary) and, where certified concentrations were below the method’s LODs, by analysing spiked quality control samples containing the target elements at equivalent concentrations of 5.0 mg/kg. Recovery was calculated by comparing the measured and expected concentrations. Trueness was considered acceptable if the deviation of the measured parameter did not exceed ±15%, while precision values were deemed acceptable if they were below 20%.

Linearity was assessed through the equations of the calibration curves. The study did not include an examination of the matrix effect since the Y solution, used as an internal standard, provided compensation. The certified Cd content in the reference sample was above the LOD of the method, allowing for direct measurement. The recovery values and standard deviations are presented in Table 2.

The reported Hg concentration (5.260 ± 0.195 mg/kg) refers to the spiked quality control sample used for method validation and not to the analysed fish samples.

For As, Hg, and Pb, the certified concentrations in the reference materials were below the method’s corresponding LODs; therefore, trueness for these elements was assessed using spiked QC samples. To assess these parameters, quality control (QC) samples were spiked with each target element at a concentration equivalent to 5.0 mg/kg in the original sample. The same internal standard was consistently applied. The overall acceptability of sample preparation was determined based on the recoveries of all measured elements falling within the acceptable range.

To further assess the measurement reliability of these elements, the recovery of the spiked QC samples was also evaluated. The “percentage of the spiked QC sample” was calculated by dividing the measured results of the spiked sample by the theoretical results (certified value + 5.0 mg/kg) and multiplying by 100. These spiked QC samples underwent the same sample preparation process as all other samples. As, Hg, and Pb, for which the certified concentrations in the reference materials were below the method’s LODs, the recovery of spiked QC samples was used as an indicator of the method’s trueness and evaluated against the acceptance criteria specified in the referenced validation guideline [43].

The limits of detection for the target elements were determined as 0.5 mg/kg for arsenic, 0.05 mg/kg for cadmium, 0.5 mg/kg for mercury, and 0.2 mg/kg for lead. These values represent the minimum concentrations of the substances that can be distinguished from the absence of the corresponding analyte.

#### 2.2.3. Data Handling and Calculations

Descriptive summary measures and all exposure and risk calculations were performed using Microsoft Excel 365 (Version 2605, Microsoft Corporation, Redmond, WA, USA). For elements with results below the limits of detection (LODs), results below the analytical limit of detection (left-censored data) were handled using a scenario approach: lower-bound (LB: <LOD = 0), middle-bound (MB: <LOD = LOD/2), and upper-bound (UB: <LOD = LOD). Unless stated otherwise, MB values are presented as the central estimate, and LB–UB ranges are provided to reflect uncertainty.

#### 2.2.4. Dietary Exposure and Non-Cancer Risk Characterisation

Dietary exposure was estimated and expressed as the estimated daily intake (EDI, µg/kg body weight [BW]/day) [40]:

EDI = (C × Cons × 1000)/BW,(1)

where C is the element concentration in fish (mg/kg wet weight [ww]), Cons is the daily fish consumption (kg/day), and BW is the body weight (kg). The factor 1000 converts mg to μg. Two adult consumption scenarios were assessed: the mean EU consumption (65.7 g/person/day, as used in the original dataset); and an EU high-consumer scenario, based on the chronic P95 fish meat consumption of 113.7 g/person/day reported by EFSA (European Food Safety Authority)’s consumption statistics (as summarised by RIVM). Children were assessed using a body weight of 23 kg and two consumption scenarios expressed on a body-weight basis (moderate 0.61 g/kg BW/day and high-consumer P95 2.43 g/kg BW/day, as summarised by RIVM from EFSA data). Default adult BW was set to 70 kg in line with EFSA default assumptions [44].

The screening-level non-cancer risk was characterised using the target hazard quotient (THQ) [41]:

THQ = EDI/TRV,(2)

where EDI is the estimated dietary daily intake expressed in μg/kg BW/day, and TRV is the toxicological reference value expressed in the same unit; likewise, the hazard index (HI) [40] was also used:

HI = THQ_iAs_ + THQ_Cd_ + THQ_Pb_, (3)

where HI was calculated as the sum of the THQ for each metal—inorganic As (iAs), Cd, and Pb. As arsenic speciation was not performed in the present study, inorganic arsenic was conservatively estimated to be 5% of the total arsenic, reflecting the predominance of organic arsenic species in marine fish and following a conservative assumption commonly applied in dietary exposure assessments [31,32]. Therefore, iAs-related exposure and risk estimates should be interpreted as screening-level estimates rather than as results based on direct arsenic speciation. The toxicological reference values (TRVs) used as denominators in the screening-level THQ calculations are presented in Table 3. Age-specific TRVs were applied for Pb, whereas the same TRVs were used for adults and children for iAs and Cd. Mercury was not included in the HI calculation because all Hg results were below the method’s LODs, preventing a quantitative exposure assessment.

## 3. Results

The individual analytical results for all 40 European seabass samples are provided in Supplementary Table S1. Among the 40 analysed European seabass samples purchased from a Hungarian retail market, total As was detected in 37/40 samples (92.5%), Cd in 14/40 samples (35.0%), and Pb in 11/40 samples (27.5%). All Hg results were below the LODs (0.50 mg/kg), which prevented the quantitative assessment of Hg concentrations below this level. Mean (±SD) concentrations in muscle tissue (mg/kg ww) were 1.44 ± 0.90 for total As, 0.06 ± 0.02 for Cd, and 0.25 ± 0.14 for Pb. Inorganic arsenic (iAs) was calculated based on the assumption that iAs constituted 5% of the total As, resulting in 0.07 ± 0.05 mg/kg ww. Considerable variability was observed for As, consistent with the known species and habitat-related differences in arsenobetaine accumulation. In contrast, Cd and Pb concentrations were generally low but showed a subset of elevated values. When compared with the EU maximum levels (MLs) established by Regulation (EU) 2023/915 [37] and Regulation (EU) 2025/1891 [38], six samples (15%) exceeded the ML for Pb (0.30 mg/kg), and 13 samples (32.5%) exceeded the ML for Cd (0.05 mg/kg for fish species specifically not listed). For iAs, eight samples (20%) potentially exceeded the ML of 0.10 mg/kg under the conservative assumption that iAs represents 5% of total As; however, because arsenic speciation was not performed, this comparison should be interpreted as an indicative rather than a formal compliance assessment. Moreover, two samples exceeded the indicative MLs for Cd and Pb, six samples exceeded the indicative MLs for iAS and Cd, and one sample exceeded the MLs for iAS, Cd, and Pb. These findings indicate that a non-negligible proportion of the analysed seabass samples exceeded current EU MLs for Cd and/or Pb, while the iAs comparison highlights the need for direct arsenic speciation in future studies.

Lower-bound (LB), middle-bound (MB), and upper-bound (UB) scenarios were applied to account for measurements below the analytical LODs. Table 4 summarises the resulting mean concentrations.

For iAs, the LB–UB range was narrow due to the assumption that iAs constitutes 5% of total As, and because total As was detected in nearly all samples. In contrast, Cd and Pb showed wider LB–UB ranges, reflecting the higher proportion of <LOD results and the influence of substitution methods on estimated central tendencies. These differences propagate into exposure and risk estimates.

Dietary exposures were estimated for adults and children using MB concentrations, with LB–UB ranges provided in brackets. Four consumption scenarios were evaluated: mean EU adult consumption, high-consuming adults (P95), moderate-consuming children, and high-consuming children (P95). Table 5 summarises the estimated daily intake (EDI, µg/kg BW/day) for iAs, Cd, and Pb; the corresponding element-specific target hazard quotient (THQ) values; and the resulting hazard index (HI). The hazard index (HI) was calculated as the sum of the individual target hazard quotient (THQ) values for the assessed elements; the individual element-specific THQ values and the resulting cumulative HIs are presented in Table 5.

Across all scenarios, Pb was the dominant driver of non-cancer risks, while iAs and Cd made smaller contributions to the overall HI. For adults, the HI exceeded one under both mean consumption (HI = 1.32) and high-consumption (HI = 2.28) scenarios, indicating a potential screening-level non-cancer health concern. For children, the moderate-consumption scenario yielded an HI below one (0.59), but the high-consumption scenario resulted in an HI of 2.36, suggesting that the frequent consumption of seabass could raise concerns under the assumptions applied.

The LB–UB ranges showed that uncertainty related to <LOD handling affects the magnitude of the estimated risk, particularly for Pb, but it does not change the classification of the high-consumer scenarios: high-consuming adults and children consistently exceeded HI = 1, while moderate-consuming children remained below this threshold. For mean adult consumptions, the interpretation was more borderline, with the LB scenario below one and the MB/UB scenarios at or above one.

## 4. Discussion

### 4.1. Occurrence and Comparison with Literature

The measured concentrations of As, Cd, and Pb in European seabass were broadly consistent with previous reports for Mediterranean and/or Adriatic European seabass and other marine fish species, although between-study comparisons should be interpreted cautiously due to differences in species, size, age, sampling location, season, and analytical methodology [13,45,46,47,48,49,50,51,52,53]. Total As was detected in nearly all samples, reflecting the well-known tendency of marine fish to accumulate organic arsenic species, such as arsenobetaine, which are considered to have a low toxicity [36,37,38]. In the absence of arsenic speciation data, iAs was estimated to be 5% of total As; therefore, the resulting iAs concentrations should be interpreted as conservative screening estimates rather than measured occurrence data.

Cd and Pb showed more variable occurrence, with a subset of samples exceeding EU maximum levels. This pattern aligns with several studies from the Adriatic and surrounding regions. Perugini et al. reported comparable Cd concentrations (0.07 mg/kg ww) in several Adriatic marine fish species, although their As levels were substantially higher and their Pb levels lower than those observed here [47]. Bilandžić et al. similarly found As, Cd, Hg, and Pb concentrations in Croatian Adriatic marine fish species that were broadly comparable to our results, with most values below regulatory limits [48].

The higher accumulation of PTEs in liver tissue is well-documented [13], but muscle concentrations remain the most relevant for consumer exposure. Several recent studies have highlighted Pb as a recurring concern in retail fish. Lehel et al. reported Pb exceedances in 40% of tuna samples sold in Hungary [46], while Plachy et al. found Pb above the EU limit in 73% of *Sardina pilchardus* samples [45]. These findings mirror the Pb exceedances observed in the present study (15%), although the exceedance rate observed here was lower than those in retail fish studies.

Other regional studies have also reported similar patterns. Sepe et al. found Cd and Pb levels in Adriatic fish below those detected here [49], while Jureša and Blanuša demonstrated that shellfish accumulated higher Cd and Pb levels than finfish [50]. Copat et al. observed elevated PTE concentrations in fish from petrochemical-impacted Mediterranean areas, though THQ values still suggested a low risk [51]. Makedonski et al. reported As, Pb, and Hg concentrations in Black Sea fish similar to those in our dataset [52]. Renieri et al. showed species-and season-dependent variation in Cd, Pb, and Hg in seabass and seabream from Aegean and Cretan aquaculture sites [53].

Taken together, the concentrations observed in the analysed retail European seabass samples fell within the broader range reported for Mediterranean fish. However, the proportion of Cd and Pb exceedances observed in these samples highlights the importance of continued surveillance and supply-chain controls for fish marketed to consumers. Importantly, direct regulatory exceedances were observed for Cd and Pb, whereas the iAs comparison was assumption-based since inorganic arsenic was not measured directly. This distinction is essential for interpretation: the Cd and Pb findings can be considered compliance-relevant, while the iAs results indicate a need for future arsenic speciation instead of providing definitive evidence of non-compliance.

The occurrence of cadmium and lead in marine fish is influenced by multiple environmental factors. In the Adriatic Sea, potentially toxic elements have been associated with anthropogenic inputs, including industrial and urban discharges, maritime traffic, port activities, riverine transport, and agricultural runoff. Their bioavailability may further be affected by local sediment characteristics and environmental conditions. Nevertheless, the present study was not designed to identify contamination sources, and these factors are provided only as a possible background for interpreting the occurrence data.

### 4.2. Handling of <LOD Results and Implications for Risk Estimates

Because a substantial proportion of Cd and Pb results were reported as <LOD, risk estimates depend on how these left-censored values are treated. Presenting LB/MB/UB scenarios avoids overconfidence in a single estimate, as LB may underestimate exposure while UB represents a conservative upper bound. Based on the results presented in Table 5, the choice of bound had the largest impact on Pb-driven hazard metrics, especially in high-consumer scenarios. This pattern is consistent with previous work demonstrating that Pb exposure estimates are highly sensitive to analytical detection limits and substitution methods [21,39,44,54,55,56,57,58,59,60].

For the mean adult consumption, the interpretation was borderline: the LB estimate remained below HI = 1, whereas the MB and UB estimates were at or above this threshold. In contrast, high-consuming adults and high-consuming children exceeded HI = 1 across LB, MB, and UB assumptions. This indicates that the high-consumer findings are more robust to left-censoring assumptions than the mean adult scenario.

### 4.3. Adult vs. Child Scenarios and the Role of Consumption Assumptions

Using age-appropriate body weights and child-specific consumption scenarios are essential for accurate consumer risk characterisation. Under mean adult consumption, the HI was 1.32 under the MB assumption, with an LB–UB range of 0.87–1.76, demonstrating that interpretation of this scenario is sensitive to the treatment of results below the LODs. In contrast, the adult high-consumer (P95) scenario resulted in an HI of 2.28 [1.51–3.05], exceeding one under all three bound assumptions. Lead was the major contributor to the cumulative hazard, with an MB THQ of 1.056 under the mean adult consumption and 1.827 under the adult P95 scenario, compared with substantially lower THQs for iAs and Cd. For children, moderate consumption resulted in an HI of 0.59 [0.41–0.77], remaining below one across all assumptions, whereas the high-consumer scenario resulted in an HI of 2.36 [1.64–3.08]. Thus, the increase in screening-level concerns under high-consumption conditions was evident in both adults and children despite the lower absolute food intake of children.

However, the consumption scenarios should not be interpreted as species-specific seabass intake. They were based on generic fish-consumption assumptions and therefore represent conservative screening scenarios in which seabass is assumed to contribute substantially to total fish intake. This is useful for identifying potential risk situations, but it may overestimate exposure for consumers who eat seabass only occasionally or as part of a varied fish diet. The calculated HI values therefore indicate a clear screening-level concern under the high-consumption scenarios, while the mean adult scenario remains dependent on the treatment of the results below the LODs, with HI below one under LB but above one under MB and UB assumptions.

### 4.4. Mercury and Analytical Sensitivity

All Hg results were below the method’s LODs, precluding quantitative dietary risk characterisation for mercury. Accordingly, Hg was not included in the HI calculations. Future work would benefit from an analytical method with an LOD well below the applicable maximum level to enable more informative occurrence estimates and trend monitoring.

Because the method’s LOD for Hg was 0.50 mg/kg, the absence of quantified Hg concentrations should not be interpreted as evidence of negligible mercury exposure. Rather, the results indicate that Hg could not be quantified below this concentration level with the analytical method applied. Consequently, no Hg-specific EDI or THQ could be derived, and Hg could not contribute quantitatively to the cumulative HI in the present assessment.

### 4.5. Interpretation of Screening-Level Risk Estimates

The THQ/HI approach applied in this study should be interpreted as a screening-level risk characterisation tool. This is particularly important for Pb and iAs. For Pb, no safe exposure threshold has yet been established; therefore, Pb-related THQ values should be interpreted as prioritisation indicators rather than as definitive evidence of adverse health effects. For iAs, the risk estimate was based on an assumed fixed proportion of total As rather than direct speciation, which introduces additional uncertainty.

The element-specific THQ results provide further context for the cumulative HI values. Across all four consumption scenarios, Pb made the largest contribution to the HI, whereas the contributions of iAs and Cd were comparatively small. Under the MB assumption, Pb alone exceeded THQ = 1 in the adult mean, adult P95, and child P95 scenarios, while remaining below one in the moderate child scenario. Consequently, the elevated cumulative HI values observed in the higher-exposure scenarios were driven predominantly by Pb rather than by a similar contribution from all assessed elements. The complete sample-specific EDI, THQ, and HI calculations under LB, MB, and UB assumptions are provided in Tables S2–S5 in the Supplementary Materials.

Overall, these results should not be interpreted as definitive evidence of a general population-level health risk. Nevertheless, the HI exceeded one in the adult mean scenario under MB and UB assumptions, as well as in both adult and child high-consumer scenarios across all LB/MB/UB assumptions. These results identify specific exposure conditions in which screening-level non-cancer concerns arise and show that the strength of this conclusion differs between scenarios: it is robust across censoring assumptions for high consumers but remains sensitive to uncertainty for mean adult consumption. The results support continued risk-based monitoring of Pb and Cd in European seabass, together with direct inorganic arsenic speciation and more sensitive mercury analysis, to reduce the remaining uncertainties identified in this screening-level assessment.

## 5. Conclusions and Interpretation of Consumer Risk

This study provides a screening-level consumer risk characterisation for potentially toxic elements in European seabass (*Dicentrarchus labrax*) marketed in Hungary. Measured concentrations demonstrated the presence of total arsenic, cadmium, and lead in edible muscle tissue, with mercury remaining below the analytical limit of detection in all samples. A subset of samples exceeded the current EU maximum levels for Cd and Pb. For iAs, exceedance was assessed only indicatively, as inorganic arsenic was not measured directly but estimated to be 5% of total arsenic.

Based on screening-level risk characterisation using the target hazard quotient (THQ) and hazard index (HI), European seabass did not provide definitive evidence of a general population-level health risk, although screening-level concerns were identified under several exposure scenarios. However, the results consistently indicated that lead was the dominant contributor to non-cancer risk, and that elevated THQ and HI values may occur under conservative assumptions, particularly under high-consumption scenarios. In the mean adult consumption scenario, the interpretation was borderline, with the HI below one under the lower-bound scenario but above one under the middle- and upper-bound scenarios. Accordingly, potential consumer risks cannot be excluded for frequent consumers and sensitive population groups.

The interpretation and generalisability of these findings require careful consideration of several limitations. Risk estimates were derived from average- and high-consumer fish-consumption scenarios rather than individual-level dietary data, and consumption values were not species-specific, implicitly assuming that European seabass constitutes a substantial share of the total fish intake. In addition, a substantial proportion of cadmium and lead results were below the analytical limits of detection; to address this uncertainty, lower-, middle-, and upper-bound scenarios were applied, which demonstrated that risk estimates—particularly for lead—were sensitive to assumptions regarding concentrations below detection limits. Inorganic arsenic was not directly measured but was conservatively estimated as a fixed proportion of total arsenic. As no species- or region-specific conversion data were available for *Dicentrarchus labrax* from the Adriatic Sea, this approach introduces additional uncertainty and represents a major limitation of the present screening-level risk assessment, particularly for a compliance-related interpretation of iAs. Finally, quantitative mercury risk characterisation was not feasible due to insufficient analytical sensitivity, though the absence of quantified mercury concentrations should not be interpreted as absence of exposure. In addition, no morphometric or physiological characteristics (e.g., fish length, weight, condition index, or hepatosomatic index) were recorded, precluding the evaluation of potential relationships between fish characteristics and potentially toxic element concentrations. The available dataset did not permit stratification of samples according to individual retail market origins. Furthermore, although the validated ICP-OES method was considered fit for purpose, its higher detection limits compared with ICP-MS may have reduced the sensitivity for quantifying ultra-low trace concentrations, particularly mercury, and consequently limited the precision of exposure estimates at very low concentration levels.

Taken together, these results should be interpreted as the identification of consumption scenarios under which screening-level non-cancer concerns may arise rather than as evidence of adverse health effects at the population level. The findings support continued monitoring of inorganic arsenic, cadmium, and lead in European seabass marketed for human consumption while emphasising the importance of risk-based communication targeted to frequent fish consumers and vulnerable groups rather than broad population-wide advisories. Future studies should confirm these screening-level findings using species-specific consumption data, direct inorganic arsenic speciation, and more sensitive mercury analytical methods, thereby enabling a more robust regulatory and consumer risk assessment.

## Figures and Tables

**Table 1:** Results of validation.

Element	Wavelength of Detection (nm)	Calibration Curve Parameters			Limit of Quantitation (mg/kg)	Limit of Detection (mg/kg)	Precision (%)	Trueness (%)
		Equation (y = a · x + b) (1)		(2)				
		a	b	r				
Arsenic	188.979	1287	0	0.999828	1.67	0.50	12.7	13.6
Cadmium	228.802	63,870	0	0.999529	0.17	0.05	8.4	−10.9
Mercury	194.168	10,030	0	1.000000	1.67	0.50	12.3	8.1
Lead	220.353	6520	0	0.999813	0.67	0.20	3.5	−8.4

(1) where ‘y’ is the signal of the target element at the given concentration level, and ‘x’ is the concentration; (2) regression coefficient.

**Table 2:** Outcomes of quality control (QC) measurements (mg/kg).

Element	Certified Value	Measured Value (Without Spike)	Measured (Spiked with QC Samples)	LOD	Percentage of the Spiked QC Sample	Recovery (%)
Arsenic	0.019	ND	5.120 ± 0.180	0.500	102.0	NA
Cadmium	0.097	0.095 ± 0.006	NA	0.050	NA	98.2
Mercury	0.005	ND	5.260 ± 0.195	0.500	105.1	NA
Lead	0.063	ND	4.890 ± 0.265	0.200	96.6	NA

ND = not detected; NA = not applicable.

**Table 3:** Toxicological reference values used for screening-level THQ calculations.

Element	Population Group	TRV * (µg/kg bw/day)	Reference
Inorganic arsenic	Adults and children	0.30	[39]
Cadmium	Adults and children	1.00	[39]
Lead	Adults	0.16	[39,44]
	Children	0.26	[39,44]

* TRV: toxicological reference value. Values were used as screening-level denominators for THQ calculations. For Pb, these values should not be interpreted as formal health-based guidance values, as no safe exposure threshold has been established; therefore, Pb-related THQ values are interpreted as screening-level indicators.

**Table 4:** Mean concentrations (mg/kg wet weight) under LB/MB/UB handling of <LOD results.

Element	LB Mean	MB Mean	UB Mean
Inorganic arsenic (5% of total As)	0.070	0.071	0.072
Cadmium	0.025	0.042	0.058
Lead	0.108	0.180	0.253

**Table 5:** EDI (µg/kg bw/day), element-specific THQ, and HI under adult and child scenarios using MB concentrations (LB–UB range in brackets).

Scenario	Cons. (g/Day)	BW (kg)	EDI iAs	EDI Cd	EDI Pb	THQ iAs	THQ Cd	THQ Pb	HI
Adult (mean EU consumption)	65.7	70	0.067 [0.066–0.068]	0.039 [0.024–0.054]	0.169 [0.101–0.237]	0.223 [0.220–0.226]	0.039 [0.024–0.054]	1.056 [0.631–1.481]	1.32 [0.87–1.76]
Adult (high consumer, P95)	113.7	70	0.116 [0.114–0.117]	0.068 [0.041–0.094]	0.292 [0.175–0.410]	0.386 [0.381–0.391]	0.068 [0.041–0.094]	1.827 [1.091–2.563]	2.28 [1.51–3.05]
Child (moderate consumption) *	14.0	23	0.043 [0.043–0.044]	0.025 [0.015–0.035]	0.110 [0.066–0.154]	0.145 [0.143–0.147]	0.025 [0.015–0.035]	0.422 [0.252–0.592]	0.59 [0.41–0.77]
Child (high consumer, P95) *	55.9	23	0.173 [0.171–0.175]	0.101 [0.062–0.141]	0.437 [0.261–0.614]	0.577 [0.569–0.585]	0.101 [0.062–0.141]	1.682 [1.005–2.360]	2.36 [1.64–3.08]

* Note: The child consumption scenarios are expressed on a body-weight basis, converting them to g/day yields ~14.0 g/day (moderate) and ~55.9 g/day (P95) for a BW = 23 kg. THQ = target hazard quotient; HI = hazard index. HI was calculated as the sum of the element-specific THQs for iAs, Cd, and Pb. Mercury was not included because all Hg results were below the method’s LODs.

## Data Availability

The original contributions and data presented in this study are included in the article/Supplementary Materials. Further inquiries can be directed to the corresponding author.

## Supplementary Materials

The following supporting information can be downloaded at: https://www.mdpi.com/article/10.3390/toxics14090767/s1, Table S1: Concentrations of potentially toxic elements in European seabass (*Dicentrarchus labrax*) collected in fish retail market in Hungary; Table S2: Sample-specific EDI, THQ and HI values for the adult mean EU consumption scenario under lower-bound (LB), middle-bound (MB) and upper-bound (UB) treatment of results below the analytical LOD; Table S3: Sample-specific EDI, THQ and HI values for the adult high-consumer (P95) scenario under lower-bound (LB), middle-bound (MB) and upper-bound (UB) treatment of results below the analytical LOD; Table S4: Sample-specific EDI, THQ and HI values for the moderate child consumption scenario under lower-bound (LB), middle-bound (MB) and upper-bound (UB) treatment of results below the analytical LOD; Table S5: Sample-specific EDI, THQ and HI values for the high-consumer child (P95) scenario under lower-bound (LB), middle-bound (MB) and upper-bound (UB) treatment of results below the analytical LOD.

## Abbreviations

The following abbreviations are used in this manuscript:

As	Arsenic
Cd	Cadmium
Pb	Lead
Hg	Mercury
iAs	Inorganic arsenic
EU	European Union
ML	Maximum level
THQ	Target hazard quotient
HI	Hazard index
PTE	Potentially toxic element
Cu	Copper
Zn	Zinc
Mn	Manganese
Ni	Nickel
EDI	Estimated daily intake
QC	Quality control
LOD	Limit of detection
LOQ	Limit of quantitation
ND	Not detected
NA	Not applicable
LB	Lower-bound
MB	Middle-bound
UB	Upper-bound
C	Concentration
Cons	Consumption
BW	Body weight
EFSA	European Food Safety Authority
TRV	Toxicological reference value
ww	Wet weight
