# Supplementary material for: Consumer Risk Characterisation of Potentially Toxic Elements in European Seabass (Dicentrarchus labrax) Marketed in Hungary
Source: Toxics. 2026 Aug 27;14(9):767. doi: 10.3390/toxics14090767 (PMC13611378; doi:10.3390/toxics14090767)
Supplement: Supplementary file 1 [file toxics-14-00767-s001.zip › toxics-4461854-supplementary.pdf]

Supplementary material for Consumer risk characterisation of potentially toxic elements in European seabass (*Dicentrarchus labrax*) marketed in Hungary

Table S1. Concentrations of potentially toxic elements in European seabass (*Dicentrarchus labrax*) collected in fish retail market in Hungary.

Sample identification	As	Cd	Hg	Pb
	mg/kg	mg/kg	mg/kg	mg/kg
F1	0.50	<0.05	<0.5	0.60
F2	1.44	<0.05	<0.5	0.24
F3	0.99	<0.05	<0.5	<0.2
F4	1.70	<0.05	<0.5	0.88
F5	1.06	<0.05	<0.5	0.23
F6	<0.5	<0.05	<0.5	0.24
F7	1.76	0.058	<0.5	0.21
F8	2.42	<0.05	<0.5	<0.2
F9	0.93	<0.05	<0.5	<0.2
F10	0.85	<0.05	<0.5	<0.2
F11	0.74	<0.05	<0.5	<0.2
F12	3.25	0.095	<0.5	0.35
F13	1.34	0.050	<0.5	<0.2
F14	0.50	<0.05	<0.5	<0.2
F15	0.89	<0.05	<0.5	<0.2
F16	0.88	0.053	<0.5	0.34
F17	1.02	<0.05	<0.5	<0.2
F18	1.51	0.051	<0.5	0.43
F19	1.14	<0.05	<0.5	0.58
F20	0.68	0.057	<0.5	0.20
F21	2.57	0.082	<0.5	<0.2
F22	4.09	0.12	<0.5	<0.2
F23	3.56	0.091	<0.5	<0.2
F24	1.12	<0.05	<0.5	<0.2
F25	2.52	0.067	<0.5	<0.2
F26	3.07	0.077	<0.5	<0.2
F27	<0.5	0.073	<0.5	<0.2
F28	2.60	0.087	<0.5	<0.2
F29	1.16	<0.05	<0.5	<0.2
F30	1.47	0.051	<0.5	<0.2
F31	1.44	<0.05	<0.5	<0.2
F32	0.93	<0.05	<0.5	<0.2
F33	0.71	<0.05	<0.5	<0.2
F34	<0.5	<0.05	<0.5	<0.2
F35	1.52	<0.05	<0.5	<0.2
F36	1.19	<0.05	<0.5	<0.2
F37	0.50	<0.05	<0.5	<0.2
F38	1.23	<0.05	<0.5	<0.2
F39	1.81	<0.05	<0.5	<0.2
F40	1.14	<0.05	<0.5	<0.2

Table S2. Sample-specific EDI, THQ and HI values for the adult mean EU consumption scenario under lower-bound (LB), middle-bound (MB) and upper-bound (UB) treatment of results below the analytical LOD.

Sample	Bound	EDI iAs	EDI Cd	EDI Pb	THQ iAs	THQ Cd	THQ Pb	HI
F1	LB	0.023	0.000	0.563	0.078	0.000	3.520	3.598
F1	MB	0.023	0.023	0.563	0.078	0.023	3.520	3.621
F1	UB	0.023	0.047	0.563	0.078	0.047	3.520	3.645
F2	LB	0.068	0.000	0.225	0.225	0.000	1.408	1.633
F2	MB	0.068	0.023	0.225	0.225	0.023	1.408	1.657
F2	UB	0.068	0.047	0.225	0.225	0.047	1.408	1.680
F3	LB	0.046	0.000	0.000	0.155	0.000	0.000	0.155
F3	MB	0.046	0.023	0.094	0.155	0.023	0.587	0.765
F3	UB	0.046	0.047	0.188	0.155	0.047	1.173	1.375
F4	LB	0.080	0.000	0.826	0.266	0.000	5.162	5.428
F4	MB	0.080	0.023	0.826	0.266	0.023	5.162	5.452
F4	UB	0.080	0.047	0.826	0.266	0.047	5.162	5.475
F5	LB	0.050	0.000	0.216	0.166	0.000	1.349	1.515
F5	MB	0.050	0.023	0.216	0.166	0.023	1.349	1.538
F5	UB	0.050	0.047	0.216	0.166	0.047	1.349	1.562
F6	LB	0.000	0.000	0.225	0.000	0.000	1.408	1.408
F6	MB	0.012	0.023	0.225	0.039	0.023	1.408	1.470
F6	UB	0.023	0.047	0.225	0.078	0.047	1.408	1.533
F7	LB	0.083	0.054	0.197	0.275	0.054	1.232	1.562
F7	MB	0.083	0.054	0.197	0.275	0.054	1.232	1.562
F7	UB	0.083	0.054	0.197	0.275	0.054	1.232	1.562
F8	LB	0.114	0.000	0.000	0.379	0.000	0.000	0.379
F8	MB	0.114	0.023	0.094	0.379	0.023	0.587	0.989
F8	UB	0.114	0.047	0.188	0.379	0.047	1.173	1.599
F9	LB	0.044	0.000	0.000	0.145	0.000	0.000	0.145
F9	MB	0.044	0.023	0.094	0.145	0.023	0.587	0.756
F9	UB	0.044	0.047	0.188	0.145	0.047	1.173	1.366
F10	LB	0.040	0.000	0.000	0.133	0.000	0.000	0.133
F10	MB	0.040	0.023	0.094	0.133	0.023	0.587	0.743
F10	UB	0.040	0.047	0.188	0.133	0.047	1.173	1.353
F11	LB	0.035	0.000	0.000	0.116	0.000	0.000	0.116
F11	MB	0.035	0.023	0.094	0.116	0.023	0.587	0.726
F11	UB	0.035	0.047	0.188	0.116	0.047	1.173	1.336
F12	LB	0.153	0.089	0.329	0.508	0.089	2.053	2.651
F12	MB	0.153	0.089	0.329	0.508	0.089	2.053	2.651
F12	UB	0.153	0.089	0.329	0.508	0.089	2.053	2.651
F13	LB	0.063	0.047	0.000	0.210	0.047	0.000	0.257
F13	MB	0.063	0.047	0.094	0.210	0.047	0.587	0.843
F13	UB	0.063	0.047	0.188	0.210	0.047	1.173	1.430
F14	LB	0.023	0.000	0.000	0.078	0.000	0.000	0.078
F14	MB	0.023	0.023	0.094	0.078	0.023	0.587	0.688
F14	UB	0.023	0.047	0.188	0.078	0.047	1.173	1.298
F15	LB	0.042	0.000	0.000	0.139	0.000	0.000	0.139
F15	MB	0.042	0.023	0.094	0.139	0.023	0.587	0.749
F15	UB	0.042	0.047	0.188	0.139	0.047	1.173	1.359
F16	LB	0.041	0.050	0.319	0.138	0.050	1.994	2.182
F16	MB	0.041	0.050	0.319	0.138	0.050	1.994	2.182
F16	UB	0.041	0.050	0.319	0.138	0.050	1.994	2.182
F17	LB	0.048	0.000	0.000	0.160	0.000	0.000	0.160
F17	MB	0.048	0.023	0.094	0.160	0.023	0.587	0.770
F17	UB	0.048	0.047	0.188	0.160	0.047	1.173	1.380
F18	LB	0.071	0.048	0.404	0.236	0.048	2.522	2.806
F18	MB	0.071	0.048	0.404	0.236	0.048	2.522	2.806
F18	UB	0.071	0.048	0.404	0.236	0.048	2.522	2.806
F19	LB	0.053	0.000	0.544	0.178	0.000	3.402	3.581
F19	MB	0.053	0.023	0.544	0.178	0.023	3.402	3.604
F19	UB	0.053	0.047	0.544	0.178	0.047	3.402	3.628
F20	LB	0.032	0.053	0.188	0.106	0.053	1.173	1.333
F20	MB	0.032	0.053	0.188	0.106	0.053	1.173	1.333
F20	UB	0.032	0.053	0.188	0.106	0.053	1.173	1.333
F21	LB	0.121	0.077	0.000	0.402	0.077	0.000	0.479
F21	MB	0.121	0.077	0.094	0.402	0.077	0.587	1.066
F21	UB	0.121	0.077	0.188	0.402	0.077	1.173	1.652

Sample	Bound	EDI iAs	EDI Cd	EDI Pb	THQ iAs	THQ Cd	THQ Pb	HI
F22	LB	0.192	0.114	0.000	0.640	0.114	0.000	0.753
F22	MB	0.192	0.114	0.094	0.640	0.114	0.587	1.340
F22	UB	0.192	0.114	0.188	0.640	0.114	1.173	1.927
F23	LB	0.167	0.085	0.000	0.557	0.085	0.000	0.642
F23	MB	0.167	0.085	0.094	0.557	0.085	0.587	1.229
F23	UB	0.167	0.085	0.188	0.557	0.085	1.173	1.816
F24	LB	0.053	0.000	0.000	0.175	0.000	0.000	0.175
F24	MB	0.053	0.023	0.094	0.175	0.023	0.587	0.785
F24	UB	0.053	0.047	0.188	0.175	0.047	1.173	1.395
F25	LB	0.118	0.063	0.000	0.394	0.063	0.000	0.457
F25	MB	0.118	0.063	0.094	0.394	0.063	0.587	1.044
F25	UB	0.118	0.063	0.188	0.394	0.063	1.173	1.630
F26	LB	0.144	0.072	0.000	0.480	0.072	0.000	0.553
F26	MB	0.144	0.072	0.094	0.480	0.072	0.587	1.139
F26	UB	0.144	0.072	0.188	0.480	0.072	1.173	1.726
F27	LB	0.000	0.069	0.000	0.000	0.069	0.000	0.069
F27	MB	0.012	0.069	0.094	0.039	0.069	0.587	0.694
F27	UB	0.023	0.069	0.188	0.078	0.069	1.173	1.320
F28	LB	0.122	0.082	0.000	0.407	0.082	0.000	0.488
F28	MB	0.122	0.082	0.094	0.407	0.082	0.587	1.075
F28	UB	0.122	0.082	0.188	0.407	0.082	1.173	1.662
F29	LB	0.054	0.000	0.000	0.181	0.000	0.000	0.181
F29	MB	0.054	0.023	0.094	0.181	0.023	0.587	0.792
F29	UB	0.054	0.047	0.188	0.181	0.047	1.173	1.402
F30	LB	0.069	0.048	0.000	0.230	0.048	0.000	0.278
F30	MB	0.069	0.048	0.094	0.230	0.048	0.587	0.864
F30	UB	0.069	0.048	0.188	0.230	0.048	1.173	1.451
F31	LB	0.068	0.000	0.000	0.225	0.000	0.000	0.225
F31	MB	0.068	0.023	0.094	0.225	0.023	0.587	0.835
F31	UB	0.068	0.047	0.188	0.225	0.047	1.173	1.445
F32	LB	0.044	0.000	0.000	0.145	0.000	0.000	0.145
F32	MB	0.044	0.023	0.094	0.145	0.023	0.587	0.756
F32	UB	0.044	0.047	0.188	0.145	0.047	1.173	1.366
F33	LB	0.033	0.000	0.000	0.111	0.000	0.000	0.111
F33	MB	0.033	0.023	0.094	0.111	0.023	0.587	0.721
F33	UB	0.033	0.047	0.188	0.111	0.047	1.173	1.331
F34	LB	0.000	0.000	0.000	0.000	0.000	0.000	0.000
F34	MB	0.012	0.023	0.094	0.039	0.023	0.587	0.649
F34	UB	0.023	0.047	0.188	0.078	0.047	1.173	1.298
F35	LB	0.071	0.000	0.000	0.238	0.000	0.000	0.238
F35	MB	0.071	0.023	0.094	0.238	0.023	0.587	0.848
F35	UB	0.071	0.047	0.188	0.238	0.047	1.173	1.458
F36	LB	0.056	0.000	0.000	0.186	0.000	0.000	0.186
F36	MB	0.056	0.023	0.094	0.186	0.023	0.587	0.796
F36	UB	0.056	0.047	0.188	0.186	0.047	1.173	1.406
F37	LB	0.023	0.000	0.000	0.078	0.000	0.000	0.078
F37	MB	0.023	0.023	0.094	0.078	0.023	0.587	0.688
F37	UB	0.023	0.047	0.188	0.078	0.047	1.173	1.298
F38	LB	0.058	0.000	0.000	0.192	0.000	0.000	0.192
F38	MB	0.058	0.023	0.094	0.192	0.023	0.587	0.802
F38	UB	0.058	0.047	0.188	0.192	0.047	1.173	1.413
F39	LB	0.085	0.000	0.000	0.283	0.000	0.000	0.283
F39	MB	0.085	0.023	0.094	0.283	0.023	0.587	0.893
F39	UB	0.085	0.047	0.188	0.283	0.047	1.173	1.503
F40	LB	0.053	0.000	0.000	0.178	0.000	0.000	0.178
F40	MB	0.053	0.023	0.094	0.178	0.023	0.587	0.788
F40	UB	0.053	0.047	0.188	0.178	0.047	1.173	1.398

Notes: EDI is expressed as µg/kg bw/day. THQ = target hazard quotient; HI = hazard index. For each sample, iAs was estimated as 5% of total As after application of the respective LB/MB/UB substitution. THQ was calculated as EDI/TRV using the toxicological reference values reported in Table 3 of the main manuscript, including a Pb TRV of 0.16 µg/kg bw/day for this population group. HI was calculated as THQiAs + THQCd + THQPb. Mercury was not included because all Hg results were below the method LOD.

Table S3. Sample-specific EDI, THQ and HI values for the adult high-consumer (P95) scenario under lower-bound (LB), middle-bound (MB) and upper-bound (UB) treatment of results below the analytical LOD.

Sample	Bound	EDI iAs	EDI Cd	EDI Pb	THQ iAs	THQ Cd	THQ Pb	HI
F1	LB	0.041	0.000	0.975	0.135	0.000	6.091	6.226
F1	MB	0.041	0.041	0.975	0.135	0.041	6.091	6.267
F1	UB	0.041	0.081	0.975	0.135	0.081	6.091	6.308
F2	LB	0.117	0.000	0.390	0.390	0.000	2.436	2.826
F2	MB	0.117	0.041	0.390	0.390	0.041	2.436	2.867
F2	UB	0.117	0.081	0.390	0.390	0.081	2.436	2.907
F3	LB	0.080	0.000	0.000	0.268	0.000	0.000	0.268
F3	MB	0.080	0.041	0.162	0.268	0.041	1.015	1.324
F3	UB	0.080	0.081	0.325	0.268	0.081	2.030	2.380
F4	LB	0.138	0.000	1.429	0.460	0.000	8.934	9.394
F4	MB	0.138	0.041	1.429	0.460	0.041	8.934	9.434
F4	UB	0.138	0.081	1.429	0.460	0.081	8.934	9.475
F5	LB	0.086	0.000	0.374	0.287	0.000	2.335	2.622
F5	MB	0.086	0.041	0.374	0.287	0.041	2.335	2.662
F5	UB	0.086	0.081	0.374	0.287	0.081	2.335	2.703
F6	LB	0.000	0.000	0.390	0.000	0.000	2.436	2.436
F6	MB	0.020	0.041	0.390	0.068	0.041	2.436	2.545
F6	UB	0.041	0.081	0.390	0.135	0.081	2.436	2.653
F7	LB	0.143	0.094	0.341	0.476	0.094	2.132	2.703
F7	MB	0.143	0.094	0.341	0.476	0.094	2.132	2.703
F7	UB	0.143	0.094	0.341	0.476	0.094	2.132	2.703
F8	LB	0.197	0.000	0.000	0.655	0.000	0.000	0.655
F8	MB	0.197	0.041	0.162	0.655	0.041	1.015	1.711
F8	UB	0.197	0.081	0.325	0.655	0.081	2.030	2.767
F9	LB	0.076	0.000	0.000	0.252	0.000	0.000	0.252
F9	MB	0.076	0.041	0.162	0.252	0.041	1.015	1.308
F9	UB	0.076	0.081	0.325	0.252	0.081	2.030	2.363
F10	LB	0.069	0.000	0.000	0.230	0.000	0.000	0.230
F10	MB	0.069	0.041	0.162	0.230	0.041	1.015	1.286
F10	UB	0.069	0.081	0.325	0.230	0.081	2.030	2.342
F11	LB	0.060	0.000	0.000	0.200	0.000	0.000	0.200
F11	MB	0.060	0.041	0.162	0.200	0.041	1.015	1.256
F11	UB	0.060	0.081	0.325	0.200	0.081	2.030	2.312
F12	LB	0.264	0.154	0.569	0.880	0.154	3.553	4.587
F12	MB	0.264	0.154	0.569	0.880	0.154	3.553	4.587
F12	UB	0.264	0.154	0.569	0.880	0.154	3.553	4.587
F13	LB	0.109	0.081	0.000	0.363	0.081	0.000	0.444
F13	MB	0.109	0.081	0.162	0.363	0.081	1.015	1.459
F13	UB	0.109	0.081	0.325	0.363	0.081	2.030	2.474
F14	LB	0.041	0.000	0.000	0.135	0.000	0.000	0.135
F14	MB	0.041	0.041	0.162	0.135	0.041	1.015	1.191
F14	UB	0.041	0.081	0.325	0.135	0.081	2.030	2.247
F15	LB	0.072	0.000	0.000	0.241	0.000	0.000	0.241
F15	MB	0.072	0.041	0.162	0.241	0.041	1.015	1.297
F15	UB	0.072	0.081	0.325	0.241	0.081	2.030	2.353
F16	LB	0.071	0.086	0.552	0.238	0.086	3.452	3.776
F16	MB	0.071	0.086	0.552	0.238	0.086	3.452	3.776
F16	UB	0.071	0.086	0.552	0.238	0.086	3.452	3.776
F17	LB	0.083	0.000	0.000	0.276	0.000	0.000	0.276
F17	MB	0.083	0.041	0.162	0.276	0.041	1.015	1.332
F17	UB	0.083	0.081	0.325	0.276	0.081	2.030	2.388
F18	LB	0.123	0.083	0.698	0.409	0.083	4.365	4.857
F18	MB	0.123	0.083	0.698	0.409	0.083	4.365	4.857
F18	UB	0.123	0.083	0.698	0.409	0.083	4.365	4.857
F19	LB	0.093	0.000	0.942	0.309	0.000	5.888	6.197
F19	MB	0.093	0.041	0.942	0.309	0.041	5.888	6.237
F19	UB	0.093	0.081	0.942	0.309	0.081	5.888	6.278
F20	LB	0.055	0.093	0.325	0.184	0.093	2.030	2.307
F20	MB	0.055	0.093	0.325	0.184	0.093	2.030	2.307
F20	UB	0.055	0.093	0.325	0.184	0.093	2.030	2.307
F21	LB	0.209	0.133	0.000	0.696	0.133	0.000	0.829
F21	MB	0.209	0.133	0.162	0.696	0.133	1.015	1.844
F21	UB	0.209	0.133	0.325	0.696	0.133	2.030	2.859

Sample	Bound	EDI iAs	EDI Cd	EDI Pb	THQ iAs	THQ Cd	THQ Pb	HI
F22	LB	0.332	0.197	0.000	1.107	0.197	0.000	1.304
F22	MB	0.332	0.197	0.162	1.107	0.197	1.015	2.319
F22	UB	0.332	0.197	0.325	1.107	0.197	2.030	3.334
F23	LB	0.289	0.148	0.000	0.964	0.148	0.000	1.112
F23	MB	0.289	0.148	0.162	0.964	0.148	1.015	2.127
F23	UB	0.289	0.148	0.325	0.964	0.148	2.030	3.142
F24	LB	0.091	0.000	0.000	0.303	0.000	0.000	0.303
F24	MB	0.091	0.041	0.162	0.303	0.041	1.015	1.359
F24	UB	0.091	0.081	0.325	0.303	0.081	2.030	2.415
F25	LB	0.205	0.109	0.000	0.682	0.109	0.000	0.791
F25	MB	0.205	0.109	0.162	0.682	0.109	1.015	1.806
F25	UB	0.205	0.109	0.325	0.682	0.109	2.030	2.821
F26	LB	0.249	0.125	0.000	0.831	0.125	0.000	0.956
F26	MB	0.249	0.125	0.162	0.831	0.125	1.015	1.971
F26	UB	0.249	0.125	0.325	0.831	0.125	2.030	2.987
F27	LB	0.000	0.119	0.000	0.000	0.119	0.000	0.119
F27	MB	0.020	0.119	0.162	0.068	0.119	1.015	1.201
F27	UB	0.041	0.119	0.325	0.135	0.119	2.030	2.284
F28	LB	0.211	0.141	0.000	0.704	0.141	0.000	0.845
F28	MB	0.211	0.141	0.162	0.704	0.141	1.015	1.860
F28	UB	0.211	0.141	0.325	0.704	0.141	2.030	2.876
F29	LB	0.094	0.000	0.000	0.314	0.000	0.000	0.314
F29	MB	0.094	0.041	0.162	0.314	0.041	1.015	1.370
F29	UB	0.094	0.081	0.325	0.314	0.081	2.030	2.426
F30	LB	0.119	0.083	0.000	0.398	0.083	0.000	0.481
F30	MB	0.119	0.083	0.162	0.398	0.083	1.015	1.496
F30	UB	0.119	0.083	0.325	0.398	0.083	2.030	2.511
F31	LB	0.117	0.000	0.000	0.390	0.000	0.000	0.390
F31	MB	0.117	0.041	0.162	0.390	0.041	1.015	1.446
F31	UB	0.117	0.081	0.325	0.390	0.081	2.030	2.501
F32	LB	0.076	0.000	0.000	0.252	0.000	0.000	0.252
F32	MB	0.076	0.041	0.162	0.252	0.041	1.015	1.308
F32	UB	0.076	0.081	0.325	0.252	0.081	2.030	2.363
F33	LB	0.058	0.000	0.000	0.192	0.000	0.000	0.192
F33	MB	0.058	0.041	0.162	0.192	0.041	1.015	1.248
F33	UB	0.058	0.081	0.325	0.192	0.081	2.030	2.304
F34	LB	0.000	0.000	0.000	0.000	0.000	0.000	0.000
F34	MB	0.020	0.041	0.162	0.068	0.041	1.015	1.123
F34	UB	0.041	0.081	0.325	0.135	0.081	2.030	2.247
F35	LB	0.123	0.000	0.000	0.411	0.000	0.000	0.411
F35	MB	0.123	0.041	0.162	0.411	0.041	1.015	1.467
F35	UB	0.123	0.081	0.325	0.411	0.081	2.030	2.523
F36	LB	0.097	0.000	0.000	0.322	0.000	0.000	0.322
F36	MB	0.097	0.041	0.162	0.322	0.041	1.015	1.378
F36	UB	0.097	0.081	0.325	0.322	0.081	2.030	2.434
F37	LB	0.041	0.000	0.000	0.135	0.000	0.000	0.135
F37	MB	0.041	0.041	0.162	0.135	0.041	1.015	1.191
F37	UB	0.041	0.081	0.325	0.135	0.081	2.030	2.247
F38	LB	0.100	0.000	0.000	0.333	0.000	0.000	0.333
F38	MB	0.100	0.041	0.162	0.333	0.041	1.015	1.389
F38	UB	0.100	0.081	0.325	0.333	0.081	2.030	2.445
F39	LB	0.147	0.000	0.000	0.490	0.000	0.000	0.490
F39	MB	0.147	0.041	0.162	0.490	0.041	1.015	1.546
F39	UB	0.147	0.081	0.325	0.490	0.081	2.030	2.602
F40	LB	0.093	0.000	0.000	0.309	0.000	0.000	0.309
F40	MB	0.093	0.041	0.162	0.309	0.041	1.015	1.364
F40	UB	0.093	0.081	0.325	0.309	0.081	2.030	2.420

Notes: EDI is expressed as µg/kg bw/day. THQ = target hazard quotient; HI = hazard index. For each sample, iAs was estimated as 5% of total As after application of the respective LB/MB/UB substitution. THQ was calculated as EDI/TRV using the toxicological reference values reported in Table 3 of the main manuscript, including a Pb TRV of 0.16 µg/kg bw/day for this population group. HI was calculated as THQiAs + THQCd + THQPb. Mercury was not included because all Hg results were below the method LOD.

Table S4. Sample-specific EDI, THQ and HI values for the moderate child consumption scenario under lower-bound (LB), middle-bound (MB) and upper-bound (UB) treatment of results below the analytical LOD.

Sample	Bound	EDI iAs	EDI Cd	EDI Pb	THQ iAs	THQ Cd	THQ Pb	HI
F1	LB	0.015	0.000	0.366	0.051	0.000	1.408	1.459
F1	MB	0.015	0.015	0.366	0.051	0.015	1.408	1.474
F1	UB	0.015	0.030	0.366	0.051	0.030	1.408	1.489
F2	LB	0.044	0.000	0.146	0.146	0.000	0.563	0.709
F2	MB	0.044	0.015	0.146	0.146	0.015	0.563	0.725
F2	UB	0.044	0.030	0.146	0.146	0.030	0.563	0.740
F3	LB	0.030	0.000	0.000	0.101	0.000	0.000	0.101
F3	MB	0.030	0.015	0.061	0.101	0.015	0.235	0.351
F3	UB	0.030	0.030	0.122	0.101	0.030	0.469	0.600
F4	LB	0.052	0.000	0.537	0.173	0.000	2.065	2.237
F4	MB	0.052	0.015	0.537	0.173	0.015	2.065	2.253
F4	UB	0.052	0.030	0.537	0.173	0.030	2.065	2.268
F5	LB	0.032	0.000	0.140	0.108	0.000	0.540	0.647
F5	MB	0.032	0.015	0.140	0.108	0.015	0.540	0.663
F5	UB	0.032	0.030	0.140	0.108	0.030	0.540	0.678
F6	LB	0.000	0.000	0.146	0.000	0.000	0.563	0.563
F6	MB	0.008	0.015	0.146	0.025	0.015	0.563	0.604
F6	UB	0.015	0.030	0.146	0.051	0.030	0.563	0.644
F7	LB	0.054	0.035	0.128	0.179	0.035	0.493	0.707
F7	MB	0.054	0.035	0.128	0.179	0.035	0.493	0.707
F7	UB	0.054	0.035	0.128	0.179	0.035	0.493	0.707
F8	LB	0.074	0.000	0.000	0.246	0.000	0.000	0.246
F8	MB	0.074	0.015	0.061	0.246	0.015	0.235	0.496
F8	UB	0.074	0.030	0.122	0.246	0.030	0.469	0.746
F9	LB	0.028	0.000	0.000	0.095	0.000	0.000	0.095
F9	MB	0.028	0.015	0.061	0.095	0.015	0.235	0.344
F9	UB	0.028	0.030	0.122	0.095	0.030	0.469	0.594
F10	LB	0.026	0.000	0.000	0.086	0.000	0.000	0.086
F10	MB	0.026	0.015	0.061	0.086	0.015	0.235	0.336
F10	UB	0.026	0.030	0.122	0.086	0.030	0.469	0.586
F11	LB	0.023	0.000	0.000	0.075	0.000	0.000	0.075
F11	MB	0.023	0.015	0.061	0.075	0.015	0.235	0.325
F11	UB	0.023	0.030	0.122	0.075	0.030	0.469	0.575
F12	LB	0.099	0.058	0.213	0.330	0.058	0.821	1.210
F12	MB	0.099	0.058	0.213	0.330	0.058	0.821	1.210
F12	UB	0.099	0.058	0.213	0.330	0.058	0.821	1.210
F13	LB	0.041	0.030	0.000	0.136	0.030	0.000	0.167
F13	MB	0.041	0.030	0.061	0.136	0.030	0.235	0.401
F13	UB	0.041	0.030	0.122	0.136	0.030	0.469	0.636
F14	LB	0.015	0.000	0.000	0.051	0.000	0.000	0.051
F14	MB	0.015	0.015	0.061	0.051	0.015	0.235	0.301
F14	UB	0.015	0.030	0.122	0.051	0.030	0.469	0.551
F15	LB	0.027	0.000	0.000	0.090	0.000	0.000	0.090
F15	MB	0.027	0.015	0.061	0.090	0.015	0.235	0.340
F15	UB	0.027	0.030	0.122	0.090	0.030	0.469	0.590
F16	LB	0.027	0.032	0.207	0.089	0.032	0.798	0.919
F16	MB	0.027	0.032	0.207	0.089	0.032	0.798	0.919
F16	UB	0.027	0.032	0.207	0.089	0.032	0.798	0.919
F17	LB	0.031	0.000	0.000	0.104	0.000	0.000	0.104
F17	MB	0.031	0.015	0.061	0.104	0.015	0.235	0.354
F17	UB	0.031	0.030	0.122	0.104	0.030	0.469	0.603
F18	LB	0.046	0.031	0.262	0.154	0.031	1.009	1.193
F18	MB	0.046	0.031	0.262	0.154	0.031	1.009	1.193
F18	UB	0.046	0.031	0.262	0.154	0.031	1.009	1.193
F19	LB	0.035	0.000	0.354	0.116	0.000	1.361	1.477
F19	MB	0.035	0.015	0.354	0.116	0.015	1.361	1.492
F19	UB	0.035	0.030	0.354	0.116	0.030	1.361	1.507
F20	LB	0.021	0.035	0.122	0.069	0.035	0.469	0.573
F20	MB	0.021	0.035	0.122	0.069	0.035	0.469	0.573
F20	UB	0.021	0.035	0.122	0.069	0.035	0.469	0.573
F21	LB	0.078	0.050	0.000	0.261	0.050	0.000	0.311
F21	MB	0.078	0.050	0.061	0.261	0.050	0.235	0.546
F21	UB	0.078	0.050	0.122	0.261	0.050	0.469	0.781

Sample	Bound	EDI iAs	EDI Cd	EDI Pb	THQ iAs	THQ Cd	THQ Pb	HI
F22	LB	0.125	0.074	0.000	0.416	0.074	0.000	0.490
F22	MB	0.125	0.074	0.061	0.416	0.074	0.235	0.724
F22	UB	0.125	0.074	0.122	0.416	0.074	0.469	0.959
F23	LB	0.109	0.056	0.000	0.362	0.056	0.000	0.417
F23	MB	0.109	0.056	0.061	0.362	0.056	0.235	0.652
F23	UB	0.109	0.056	0.122	0.362	0.056	0.469	0.887
F24	LB	0.034	0.000	0.000	0.114	0.000	0.000	0.114
F24	MB	0.034	0.015	0.061	0.114	0.015	0.235	0.364
F24	UB	0.034	0.030	0.122	0.114	0.030	0.469	0.614
F25	LB	0.077	0.041	0.000	0.256	0.041	0.000	0.297
F25	MB	0.077	0.041	0.061	0.256	0.041	0.235	0.532
F25	UB	0.077	0.041	0.122	0.256	0.041	0.469	0.766
F26	LB	0.094	0.047	0.000	0.312	0.047	0.000	0.359
F26	MB	0.094	0.047	0.061	0.312	0.047	0.235	0.594
F26	UB	0.094	0.047	0.122	0.312	0.047	0.469	0.828
F27	LB	0.000	0.045	0.000	0.000	0.045	0.000	0.045
F27	MB	0.008	0.045	0.061	0.025	0.045	0.235	0.305
F27	UB	0.015	0.045	0.122	0.051	0.045	0.469	0.565
F28	LB	0.079	0.053	0.000	0.264	0.053	0.000	0.317
F28	MB	0.079	0.053	0.061	0.264	0.053	0.235	0.552
F28	UB	0.079	0.053	0.122	0.264	0.053	0.469	0.787
F29	LB	0.035	0.000	0.000	0.118	0.000	0.000	0.118
F29	MB	0.035	0.015	0.061	0.118	0.015	0.235	0.368
F29	UB	0.035	0.030	0.122	0.118	0.030	0.469	0.618
F30	LB	0.045	0.031	0.000	0.149	0.031	0.000	0.181
F30	MB	0.045	0.031	0.061	0.149	0.031	0.235	0.415
F30	UB	0.045	0.031	0.122	0.149	0.031	0.469	0.650
F31	LB	0.044	0.000	0.000	0.146	0.000	0.000	0.146
F31	MB	0.044	0.015	0.061	0.146	0.015	0.235	0.396
F31	UB	0.044	0.030	0.122	0.146	0.030	0.469	0.646
F32	LB	0.028	0.000	0.000	0.095	0.000	0.000	0.095
F32	MB	0.028	0.015	0.061	0.095	0.015	0.235	0.344
F32	UB	0.028	0.030	0.122	0.095	0.030	0.469	0.594
F33	LB	0.022	0.000	0.000	0.072	0.000	0.000	0.072
F33	MB	0.022	0.015	0.061	0.072	0.015	0.235	0.322
F33	UB	0.022	0.030	0.122	0.072	0.030	0.469	0.572
F34	LB	0.000	0.000	0.000	0.000	0.000	0.000	0.000
F34	MB	0.008	0.015	0.061	0.025	0.015	0.235	0.275
F34	UB	0.015	0.030	0.122	0.051	0.030	0.469	0.551
F35	LB	0.046	0.000	0.000	0.155	0.000	0.000	0.155
F35	MB	0.046	0.015	0.061	0.155	0.015	0.235	0.404
F35	UB	0.046	0.030	0.122	0.155	0.030	0.469	0.654
F36	LB	0.036	0.000	0.000	0.121	0.000	0.000	0.121
F36	MB	0.036	0.015	0.061	0.121	0.015	0.235	0.371
F36	UB	0.036	0.030	0.122	0.121	0.030	0.469	0.621
F37	LB	0.015	0.000	0.000	0.051	0.000	0.000	0.051
F37	MB	0.015	0.015	0.061	0.051	0.015	0.235	0.301
F37	UB	0.015	0.030	0.122	0.051	0.030	0.469	0.551
F38	LB	0.038	0.000	0.000	0.125	0.000	0.000	0.125
F38	MB	0.038	0.015	0.061	0.125	0.015	0.235	0.375
F38	UB	0.038	0.030	0.122	0.125	0.030	0.469	0.625
F39	LB	0.055	0.000	0.000	0.184	0.000	0.000	0.184
F39	MB	0.055	0.015	0.061	0.184	0.015	0.235	0.434
F39	UB	0.055	0.030	0.122	0.184	0.030	0.469	0.684
F40	LB	0.035	0.000	0.000	0.116	0.000	0.000	0.116
F40	MB	0.035	0.015	0.061	0.116	0.015	0.235	0.366
F40	UB	0.035	0.030	0.122	0.116	0.030	0.469	0.616

Notes: EDI is expressed as µg/kg bw/day. THQ = target hazard quotient; HI = hazard index. For each sample, iAs was estimated as 5% of total As after application of the respective LB/MB/UB substitution. THQ was calculated as EDI/TRV using the toxicological reference values reported in Table 3 of the main manuscript, including a Pb TRV of 0.26 µg/kg bw/day for this population group. HI was calculated as THQiAs + THQCd + THQPb. Mercury was not included because all Hg results were below the method LOD.

Table S5. Sample-specific EDI, THQ and HI values for the high-consumer child (P95) scenario under lower-bound (LB), middle-bound (MB) and upper-bound (UB) treatment of results below the analytical LOD.

Sample	Bound	EDI iAs	EDI Cd	EDI Pb	THQ iAs	THQ Cd	THQ Pb	HI
F1	LB	0.061	0.000	1.458	0.203	0.000	5.608	5.810
F1	MB	0.061	0.061	1.458	0.203	0.061	5.608	5.871
F1	UB	0.061	0.122	1.458	0.203	0.122	5.608	5.932
F2	LB	0.175	0.000	0.583	0.583	0.000	2.243	2.826
F2	MB	0.175	0.061	0.583	0.583	0.061	2.243	2.887
F2	UB	0.175	0.122	0.583	0.583	0.122	2.243	2.948
F3	LB	0.120	0.000	0.000	0.401	0.000	0.000	0.401
F3	MB	0.120	0.061	0.243	0.401	0.061	0.935	1.396
F3	UB	0.120	0.122	0.486	0.401	0.122	1.869	2.392
F4	LB	0.207	0.000	2.138	0.689	0.000	8.225	8.913
F4	MB	0.207	0.061	2.138	0.689	0.061	8.225	8.974
F4	UB	0.207	0.122	2.138	0.689	0.122	8.225	9.035
F5	LB	0.129	0.000	0.559	0.429	0.000	2.150	2.579
F5	MB	0.129	0.061	0.559	0.429	0.061	2.150	2.640
F5	UB	0.129	0.122	0.559	0.429	0.122	2.150	2.700
F6	LB	0.000	0.000	0.583	0.000	0.000	2.243	2.243
F6	MB	0.030	0.061	0.583	0.101	0.061	2.243	2.405
F6	UB	0.061	0.122	0.583	0.203	0.122	2.243	2.567
F7	LB	0.214	0.141	0.510	0.713	0.141	1.963	2.816
F7	MB	0.214	0.141	0.510	0.713	0.141	1.963	2.816
F7	UB	0.214	0.141	0.510	0.713	0.141	1.963	2.816
F8	LB	0.294	0.000	0.000	0.980	0.000	0.000	0.980
F8	MB	0.294	0.061	0.243	0.980	0.061	0.935	1.975
F8	UB	0.294	0.122	0.486	0.980	0.122	1.869	2.971
F9	LB	0.113	0.000	0.000	0.377	0.000	0.000	0.377
F9	MB	0.113	0.061	0.243	0.377	0.061	0.935	1.372
F9	UB	0.113	0.122	0.486	0.377	0.122	1.869	2.367
F10	LB	0.103	0.000	0.000	0.344	0.000	0.000	0.344
F10	MB	0.103	0.061	0.243	0.344	0.061	0.935	1.340
F10	UB	0.103	0.122	0.486	0.344	0.122	1.869	2.335
F11	LB	0.090	0.000	0.000	0.300	0.000	0.000	0.300
F11	MB	0.090	0.061	0.243	0.300	0.061	0.935	1.295
F11	UB	0.090	0.122	0.486	0.300	0.122	1.869	2.290
F12	LB	0.395	0.231	0.851	1.316	0.231	3.271	4.818
F12	MB	0.395	0.231	0.851	1.316	0.231	3.271	4.818
F12	UB	0.395	0.231	0.851	1.316	0.231	3.271	4.818
F13	LB	0.163	0.122	0.000	0.543	0.122	0.000	0.664
F13	MB	0.163	0.122	0.243	0.543	0.122	0.935	1.599
F13	UB	0.163	0.122	0.486	0.543	0.122	1.869	2.533
F14	LB	0.061	0.000	0.000	0.203	0.000	0.000	0.203
F14	MB	0.061	0.061	0.243	0.203	0.061	0.935	1.198
F14	UB	0.061	0.122	0.486	0.203	0.122	1.869	2.193
F15	LB	0.108	0.000	0.000	0.360	0.000	0.000	0.360
F15	MB	0.108	0.061	0.243	0.360	0.061	0.935	1.356
F15	UB	0.108	0.122	0.486	0.360	0.122	1.869	2.351
F16	LB	0.107	0.129	0.826	0.356	0.129	3.178	3.663
F16	MB	0.107	0.129	0.826	0.356	0.129	3.178	3.663
F16	UB	0.107	0.129	0.826	0.356	0.129	3.178	3.663
F17	LB	0.124	0.000	0.000	0.413	0.000	0.000	0.413
F17	MB	0.124	0.061	0.243	0.413	0.061	0.935	1.408
F17	UB	0.124	0.122	0.486	0.413	0.122	1.869	2.404
F18	LB	0.183	0.124	1.045	0.612	0.124	4.019	4.754
F18	MB	0.183	0.124	1.045	0.612	0.124	4.019	4.754
F18	UB	0.183	0.124	1.045	0.612	0.124	4.019	4.754
F19	LB	0.139	0.000	1.409	0.462	0.000	5.421	5.882
F19	MB	0.139	0.061	1.409	0.462	0.061	5.421	5.943
F19	UB	0.139	0.122	1.409	0.462	0.122	5.421	6.004
F20	LB	0.083	0.139	0.486	0.275	0.139	1.869	2.283
F20	MB	0.083	0.139	0.486	0.275	0.139	1.869	2.283
F20	UB	0.083	0.139	0.486	0.275	0.139	1.869	2.283
F21	LB	0.312	0.199	0.000	1.041	0.199	0.000	1.240
F21	MB	0.312	0.199	0.243	1.041	0.199	0.935	2.175
F21	UB	0.312	0.199	0.486	1.041	0.199	1.869	3.109

Sample	Bound	EDI iAs	EDI Cd	EDI Pb	THQ iAs	THQ Cd	THQ Pb	HI
F22	LB	0.497	0.294	0.000	1.656	0.294	0.000	1.950
F22	MB	0.497	0.294	0.243	1.656	0.294	0.935	2.885
F22	UB	0.497	0.294	0.486	1.656	0.294	1.869	3.820
F23	LB	0.433	0.221	0.000	1.442	0.221	0.000	1.663
F23	MB	0.433	0.221	0.243	1.442	0.221	0.935	2.598
F23	UB	0.433	0.221	0.486	1.442	0.221	1.869	3.532
F24	LB	0.136	0.000	0.000	0.454	0.000	0.000	0.454
F24	MB	0.136	0.061	0.243	0.454	0.061	0.935	1.449
F24	UB	0.136	0.122	0.486	0.454	0.122	1.869	2.444
F25	LB	0.306	0.163	0.000	1.021	0.163	0.000	1.183
F25	MB	0.306	0.163	0.243	1.021	0.163	0.935	2.118
F25	UB	0.306	0.163	0.486	1.021	0.163	1.869	3.053
F26	LB	0.373	0.187	0.000	1.243	0.187	0.000	1.430
F26	MB	0.373	0.187	0.243	1.243	0.187	0.935	2.365
F26	UB	0.373	0.187	0.486	1.243	0.187	1.869	3.300
F27	LB	0.000	0.177	0.000	0.000	0.177	0.000	0.177
F27	MB	0.030	0.177	0.243	0.101	0.177	0.935	1.213
F27	UB	0.061	0.177	0.486	0.203	0.177	1.869	2.249
F28	LB	0.316	0.211	0.000	1.053	0.211	0.000	1.264
F28	MB	0.316	0.211	0.243	1.053	0.211	0.935	2.199
F28	UB	0.316	0.211	0.486	1.053	0.211	1.869	3.134
F29	LB	0.141	0.000	0.000	0.470	0.000	0.000	0.470
F29	MB	0.141	0.061	0.243	0.470	0.061	0.935	1.465
F29	UB	0.141	0.122	0.486	0.470	0.122	1.869	2.461
F30	LB	0.179	0.124	0.000	0.595	0.124	0.000	0.719
F30	MB	0.179	0.124	0.243	0.595	0.124	0.935	1.654
F30	UB	0.179	0.124	0.486	0.595	0.124	1.869	2.589
F31	LB	0.175	0.000	0.000	0.583	0.000	0.000	0.583
F31	MB	0.175	0.061	0.243	0.583	0.061	0.935	1.579
F31	UB	0.175	0.122	0.486	0.583	0.122	1.869	2.574
F32	LB	0.113	0.000	0.000	0.377	0.000	0.000	0.377
F32	MB	0.113	0.061	0.243	0.377	0.061	0.935	1.372
F32	UB	0.113	0.122	0.486	0.377	0.122	1.869	2.367
F33	LB	0.086	0.000	0.000	0.288	0.000	0.000	0.288
F33	MB	0.086	0.061	0.243	0.288	0.061	0.935	1.283
F33	UB	0.086	0.122	0.486	0.288	0.122	1.869	2.278
F34	LB	0.000	0.000	0.000	0.000	0.000	0.000	0.000
F34	MB	0.030	0.061	0.243	0.101	0.061	0.935	1.097
F34	UB	0.061	0.122	0.486	0.203	0.122	1.869	2.193
F35	LB	0.185	0.000	0.000	0.616	0.000	0.000	0.616
F35	MB	0.185	0.061	0.243	0.616	0.061	0.935	1.611
F35	UB	0.185	0.122	0.486	0.616	0.122	1.869	2.606
F36	LB	0.145	0.000	0.000	0.482	0.000	0.000	0.482
F36	MB	0.145	0.061	0.243	0.482	0.061	0.935	1.477
F36	UB	0.145	0.122	0.486	0.482	0.122	1.869	2.473
F37	LB	0.061	0.000	0.000	0.203	0.000	0.000	0.203
F37	MB	0.061	0.061	0.243	0.203	0.061	0.935	1.198
F37	UB	0.061	0.122	0.486	0.203	0.122	1.869	2.193
F38	LB	0.149	0.000	0.000	0.498	0.000	0.000	0.498
F38	MB	0.149	0.061	0.243	0.498	0.061	0.935	1.494
F38	UB	0.149	0.122	0.486	0.498	0.122	1.869	2.489
F39	LB	0.220	0.000	0.000	0.733	0.000	0.000	0.733
F39	MB	0.220	0.061	0.243	0.733	0.061	0.935	1.728
F39	UB	0.220	0.122	0.486	0.733	0.122	1.869	2.724
F40	LB	0.139	0.000	0.000	0.462	0.000	0.000	0.462
F40	MB	0.139	0.061	0.243	0.462	0.061	0.935	1.457
F40	UB	0.139	0.122	0.486	0.462	0.122	1.869	2.452

Notes: EDI is expressed as µg/kg bw/day. THQ = target hazard quotient; HI = hazard index. For each sample, iAs was estimated as 5% of total As after application of the respective LB/MB/UB substitution. THQ was calculated as EDI/TRV using the toxicological reference values reported in Table 3 of the main manuscript, including a Pb TRV of 0.26 µg/kg bw/day for this population group. HI was calculated as THQiAs + THQCd + THQPb. Mercury was not included because all Hg results were below the method LOD.
